# Evolution of Innate Immunity: Clues from Invertebrates via Fish to Mammals

**DOI:** 10.3389/fimmu.2014.00459

**Published:** 2014-09-23

**Authors:** Kurt Buchmann

**Affiliations:** ^1^Department of Veterinary Disease Biology, Faculty of Health and Medical Sciences, University of Copenhagen, Copenhagen, Denmark

**Keywords:** evolution, immunity, innate immunity, adaptive immunity, invertebrates, vertebrates

## Abstract

Host responses against invading pathogens are basic physiological reactions of all living organisms. Since the appearance of the first eukaryotic cells, a series of defense mechanisms have evolved in order to secure cellular integrity, homeostasis, and survival of the host. Invertebrates, ranging from protozoans to metazoans, possess cellular receptors, which bind to foreign elements and differentiate self from non-self. This ability is in multicellular animals associated with presence of phagocytes, bearing different names (amebocytes, hemocytes, coelomocytes) in various groups including animal sponges, worms, cnidarians, mollusks, crustaceans, chelicerates, insects, and echinoderms (sea stars and urchins). Basically, these cells have a macrophage-like appearance and function and the repair and/or fight functions associated with these cells are prominent even at the earliest evolutionary stage. The cells possess pathogen recognition receptors recognizing pathogen-associated molecular patterns, which are well-conserved molecular structures expressed by various pathogens (virus, bacteria, fungi, protozoans, helminths). Scavenger receptors, Toll-like receptors, and Nod-like receptors (NLRs) are prominent representatives within this group of host receptors. Following receptor–ligand binding, signal transduction initiates a complex cascade of cellular reactions, which lead to production of one or more of a wide array of effector molecules. Cytokines take part in this orchestration of responses even in lower invertebrates, which eventually may result in elimination or inactivation of the intruder. Important innate effector molecules are oxygen and nitrogen species, antimicrobial peptides, lectins, fibrinogen-related peptides, leucine rich repeats (LRRs), pentraxins, and complement-related proteins. Echinoderms represent the most developed invertebrates and the bridge leading to the primitive chordates, cephalochordates, and urochordates, in which many autologous genes and functions from their ancestors can be found. They exhibit numerous variants of innate recognition and effector molecules, which allow fast and innate responses toward diverse pathogens despite lack of adaptive responses. The primitive vertebrates (agnathans also termed jawless fish) were the first to supplement innate responses with adaptive elements. Thus hagfish and lampreys use LRRs as variable lymphocyte receptors, whereas higher vertebrates [cartilaginous and bony fishes (jawed fish), amphibians, reptiles, birds, and mammals] developed the major histocompatibility complex, T-cell receptors, and B-cell receptors (immunoglobulins) as additional adaptive weaponry to assist innate responses. Extensive cytokine networks are recognized in fish, but related signal molecules can be traced among invertebrates. The high specificity, antibody maturation, immunological memory, and secondary responses of adaptive immunity were so successful that it allowed higher vertebrates to reduce the number of variants of the innate molecules originating from both invertebrates and lower vertebrates. Nonetheless, vertebrates combine the two arms in an intricate inter-dependent network. Organisms at all developmental stages have, in order to survive, applied available genes and functions of which some may have been lost or may have changed function through evolution. The molecular mechanisms involved in evolution of immune molecules, might apart from simple base substitutions be as diverse as gene duplication, deletions, alternative splicing, gene recombination, domain shuffling, retrotransposition, and gene conversion. Further, variable regulation of gene expression may have played a role.

## Introduction

Host responses against invading pathogens are basic physiological reactions of all living organisms. Even prokaryotes protect themselves by use of restriction enzymes and clustered regularly interspaced palindromic repeats (CRISPRs), being able to degrade invading foreign pathogens (1). Since the appearance of the first eukaryotic cells, a series of additional defense mechanisms have evolved in order to secure cellular integrity, homeostasis, and survival of the host. Unicellular amebae developed the ability to phagocytose foreign material as a part of their food uptake mechanisms (2) and this basic phagocyte function is conserved in higher invertebrates and vertebrates in which the immunological function is more evident. Discrimination between self and non-self is also crucial for sexual functions securing genetic variation by exchange of genes between members of the same species. Recognition of non-self in both unicellular and multicellular organisms is based on cellular receptors allowing the host organism to bind, engulf, and/or kill potential invaders and offenders (3). Among the invertebrates, important groups such as protozoans (amebae, flagellates, and ciliates), sponges (such as bath sponges), cnidarians (e.g., jellyfish), worms (e.g., platyhelminths, annelids, and nematodes), mollusks (snails and bivalves), crustaceans (e.g., crabs and prawns), chelicerates (spiders, mites), insects (e.g., flies), and echinoderms (sea stars and sea urchins), are known to possess cells with receptors, which bind to foreign elements and allow differentiation of self and non-self (4). This ability is associated with presence of phagocytes bearing different names in various groups (amebocytes, hemocytes, coelomocytes, granulocytes, monocytes, macrophages), but basically they have a macrophage-like appearance and have, to a certain extent, comparable functions (5–7). Chordate evolution was based on the usage of existing genomes from ancestors and although deletions of significant parts of these have occurred, it is possible to trace some main lines from early and primitive organisms to highly developed mammals. The most primitive chordates comprising acranians (*Amphioxus*) (8–10) and tunicates (ascidians) (11) display a wide array of innate immune functions. In the primitive vertebrates comprising jawless fish (agnathans such as hagfish and lampreys), these functions became combined with an extensive use of leucine rich repeats (LRRs) as lymphocyte receptors (12, 13). With the advent of cartilaginous and bony fish, the adaptive armament [major histocompatibility complex (MHC), immunoglobulins, T-cell receptors, extensive cytokine networks] appeared, and these new tools were further developed to a high level of sophistication through amphibians, reptiles, and birds to mammals (14). This allowed a reduction of the copy number of many innate immune genes, but still the innate effector molecules have been taken into a complex network combining the obvious talents of fast acting ancient molecules with the highly developed specific recognition with memory seen in adaptive immunity. The main outlines of these aspects, which are presented below, highlight how innate immune responses evolved from ancient precursors and still play a vital and basic role even in higher vertebrates where adaptive elements are so prominent.

## The Time Scale – In Brief

**Figure 1:**
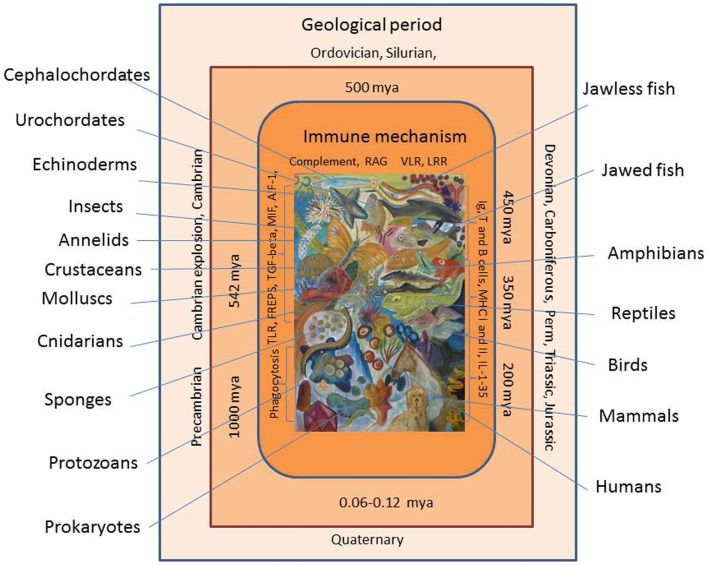
**Evolution of the immune system is shown**. Immune cells and molecules from early invertebrates to vertebrates are shown. Geological periods and time periods (million years ago, mya) are shown with extant representatives of animal groups appearing at different time during evolution.

Evolution of the animal immune system, in its broadest sense, can be viewed over a time span of at least 1000 million years (Figure 1). The age of the Earth has been estimated to more than 4.6 billion years, but the first traces of life appeared later with the appearance of primitive prokaryotes. The initial relatively inactive period is called the Precambrian period (or Proteozoic era), and it exhibited a series of primitive single celled organisms, which could exist in colonies, toward its end (one billion years ago). However, even these primitive organisms may have developed defense mechanisms to preserve their integrity. The environmental conditions prevailing then and at later stages during the Earth’s life may have placed a strong selective pressure on the organisms. Major extinctions of existing organisms (seen several times during evolution) may be due to harsh environmental and physiochemical changes, which probably have played an active role in creation of mutations, gene and chromosome deletions, duplications, and gene shuffling. The Paleozoic era spanning the period 542–240 million years ago (mya) was initiated by a new period called Cambrium 542 mya. At this stage, more complex organisms such as cnidarians (including jellyfish) were prevalent but an impressive diversification, called the Cambrian explosion or radiation, was put in action, which resulted in appearance of some major animal groups. Then over a relatively short time span, the ancestors of both invertebrates and vertebrates known today appeared. The diversification of all the multicellular animals continued. During the following, millions of years called the Ordovician, Silurian, and Devonian periods more advanced invertebrates (echinoderms), chordates (ascidians and acranians), and vertebrates (jawless and jawed vertebrates) came into play. Thus, in this last period, jawed fishes (and thereby the adaptive immune system) were seen for the first time around 450 mya and they were soon followed by amphibians. In the Carboniferous period (from around 350 mya), the reptiles appeared and diversified in the Permian period (from about 300 mya). By the end of this period, a major extinction affecting parts of all animal groups occurred probably due to some major climate changes. With the advent of the Mesozoic era initiated with the Triassic period (250–200 mya), the first dinosaurs and mammals were seen. In the Jurassic period 200–140 mya, dinosaurs radiated and birds appeared as one lineage in this group. In the following Cretaceous period (140–65 mya), the first primates developed, but again a major extinction process occurred, which primarily known as the end of the dinosaur time span. This event was followed by the Cenozoic era including the Paleogene and Neogene periods where further mammalian diversification took place and finally, in the Quaternary period, humans arose around 60,000–120,000 years ago. When dealing with innate immune mechanisms, it is thus likely that some genes involved in the defense of the early invertebrate ancestors 5–600 mya are still playing a role in the innate and even adaptive immune reactions of mammals. As will be suggested from the report below, invertebrate genes (immune-related or not) may have been used as bricks directly or modified for later and alternative use when appropriate.

## Discrimination of Self from Non-Self

Even the most primitive unicellular organism needs to discriminate self from non-self. This applies for a basic nutrition and feeding process in which the ameba or flagellate select food items and subsequently exert phagocytosis or pinocytosis. In addition, genetic exchange and sexual reproduction is dependent on this type of discrimination. It may have arisen several times during evolution but genetic evidence points to a conservation of several genes encoding molecules active in cell to cell interaction. The primitive cnidarian *Hydractinia* has at least two innate histocompatibility loci alr1 and alr2 (15). Allorecognition and rejection has been well studied for the colonial tunicate *Botryllus schlosseri* applying a locus called FuHC (fusion/histocompatibility) associated with putative receptor proteins named fester and Uncle fester, which are very polymorphic (16) and it was recently reported that a polymorphic HSP40-like protein is encoded within the FuHC locus (17). The MHC, a central element in adaptive responses, is well established in fish but its origin in invertebrates is still enigmatic. A common ancestral region traced in the early chordates (urochordates and cephalochordates) is referred to as the proto-MHC. It is likely to be the first building block for the MHC, which probably was established later in evolution by the process of chromosome duplications (18).

## Effector Cells

The basic phagocytic ability of unicellular organisms (e.g., amebae) is also found in the most primitive multicellular animals belonging to the group Porifera (sponges) and cnidarians (the group including jellyfish and sea-anemones). These animals apply phagocytic amebocytes for nutrition and recognition of foreign elements in the environment. Similar cell types have been conserved through evolution as they are recognized in all groups from invertebrates (annelids, arthropods, mollusks, echinoderms) to vertebrates (4). Several terms have been assigned to these cells in various groups and it must be expected that future investigations will sub-divide groups further. Sponges carry amebocytes in their mesoglea, cnidarians possess interstitial cells with a phagocytic function, hemocytes are found in the vascular system, and coelomocytes occur in coelomate animals. Thus, earthworms possess several subtypes of coelomocytes including eleocytes, and granular amebocytes (5) and in arthropods, comprising both crustaceans and insects, several effector cell types have been characterized (19). The evolutionary importance of corresponding phagocytes/macrophages is reflected in the range of subsets described from invertebrates and primitive chordates. Various cell types within this theme are found in advanced invertebrates (represented by echinoderms such as sea stars and sea urchins) and in the cephalochordate *Branchiostoma* (*Amphioxus)* and in urochordates (tunicates, ascidians) where both granulocyte-like cells and macrophages occur (20, 21). An even more diverse array of cell types and subsets occur in jawless vertebrates (hagfish and lampreys), cartilaginous fish (sharks and rays), and in bony fish. Besides phagocytes, jawless fish possess different subsets of lymphocytes with special membrane receptors. These primitive vertebrates without jaws have evolved an alternative antigen recognition system, which are composed of LRRs. These molecules provide agnathans a basis for establishing various lymphocyte lines corresponding to B and T lymphocytes. However, in cartilaginous and bony fish, the lymphocyte receptors are immunoglobulin (B-cell receptors) or T-cell receptors whereas agnathans apply at least two forms of variable lymphocyte receptor (VLR) based on LRR (13).

In bony fish, the cellular armament might include lymphocytes, macrophages, monocytes, dendritic cells, neutrophils, granulocytes, eosinophils, basophils, mast cells, and NK-cells and an even higher specialization is known in mammals (6, 7, 22). Leukocytes have traditionally been divided into the myeloid and lymphoid line based on their development from certain stem cells. However, B-lymphocytes in rainbow trout have been shown to exert phagocytosis (23), which suggests that the border between these developmental cell lines is less rigid at least in fish. In this context, it is interesting that the Ikaros multigene family, which take part in vertebrate hemopoietic stem cell differentiation and production of B, T and NK cell lineages, seems to find an early version in the most primitive vertebrates (the agnathan hagfish *Myxine*) and the even earlier urochordates (the tunicate *Oikopleura*) (24). The ancient origin of genes, which are central in cellular adaptive immunity in higher vertebrates, is also reflected by the finding of a Nuclear Factor of Activated T-cells (NFAT)-like gene in the primitive chordate *Branchiostoma belcheri* (*Amphioxus* group). In this chordate, this gene seems to play a role in innate recognition of lipopolysaccharide (LPS).

## Receptors

In order to respond to non-self and potential pathogens and initiate phagocytosis or production of killing mechanisms, the phagocytic cells must possess receptors, which can bind relevant ligands. The primitive multicellular sponges possess LPS binding receptors, which can interact with structural polysaccharides (beta-glucan) from fungi (25). This group has also been reported to express intracellular receptors nucleotide-binding domain and LRR (NLR) (26) (also termed the nucleotide-binding oligomerization domain receptors, Nod-like receptors), which bind bacterial or viral RNA, flagellin, and peptidoglycan leaving the host cell with an ability to fight pathogens or pathogen-related molecules, which have managed to enter the cytosol (26). RIG-like receptors (RLR) are able to bind viral RNA and establish innate defense reactions and their ancestral form seems to occur shortly before the first vertebrates evolved (27). These are all examples of pathogen-recognition receptors (PRRs) recognizing pathogen-associated molecular patterns (PAMPs), which are well-conserved molecular structures expressed by various pathogens (virus, bacteria, fungi, protozoans, helminths). PAMPs may among others be LPS, peptidoglycans, flagellin, double-strand RNA (dsRNA), and structural carbohydrates. The term damage-associated molecular patterns (DAMPs) are being used to signify the danger reflected by presence of cell constituents released to the extracellular milieu following tissue injury. Toll-like receptors (TLRs) play a major role within this group of host receptors. They are composed of an extracellular domain bearing LRRs and a cytoplasmic domain (interleukin-1 receptor like). Following receptor–ligand binding, signal transduction initiates a complex cascade of reactions, which leads to production of one or more of a wide array of effector molecules eventually resulting in elimination or inactivation of the intruder. A large number of TLRs are known with individual affinities to various PAMPs (28). TLRs have been traced to the most ancient multicellular invertebrates such as sponges, cnidarians (29), oligochaetes (earthworms) (30), mollusks (snails and mussels) (31), crustaceans (e.g., shrimps), and insects (32). The echinoderms, representing the most developed invertebrates, exhibit a complex and rich array of innate recognition molecules where among TLRs are present in numerous copies (33). The most primitive fish, the agnathans, have at least 7 identified TLRs, bony fish at least 18, amphibians 14, birds 10, and mammals 13 (28). One major receptor group comprises the scavenger receptors binding bacteria and a range of antigens including lipoproteins, which are polyanionic (34). They are ancient receptors occurring on most cells in sponges, the most primitive multicellular animals. They have a cysteine rich domain (SRCR), which can be traced through insects, echinoderms, early chordates, and fish (35).

## Effector Molecules

Invertebrates exhibit a rich variation of innate immune molecules allowing recognition, pathogen binding, and pathogen killing (16). Sponges apply oxidative killing processes based on production of reactive O- (ROS) and N-(NOS) species. Gastropods (snails) exemplified by *Biomphalaria glabrata* are able to produce ROS when exposed to one or more carbohydrate ligands (36) and NOS when infected by sporocysts of the digenean trematode *Schistosoma mansoni* (37). It is not clear if these animals possess preformed molecules (or enzymes), which are released immediately upon stimulation in order to exert their function instantly. Agglutination, clotting, and coagulation are other effective methods used to inactivate, and combat intruders and mollusks apply fibrinogen-related peptides (FREPS) as central players in the process. Melanization is another innate response mechanism in which pathogens are encapsulated and inactivated by reaction products including cytotoxic quinones and reactive O- and N-species. Melanin itself may protect against light and ionization and the prophenoloxidase system is an enzyme complex associated with these reactions (3). Many similar mechanisms have been extensively studied in fish in which inducible NO synthase is readily expressed following parasite infection (38). Other innate factors produced by fish include antimicrobial peptides (AMP), lysozyme, hemolysins, transferrins, lectins (MBL), SAA, SAP, CRP, and complement factors (39). The complement system, which is linking innate and adaptive responses in vertebrates, can be traced even in primitive invertebrates such as cnidarians (40) but exhibit the most complex cascade reactions in vertebrates. The function and interactions between the individual complement factors in lower chordates and invertebrates are unexplored and probably differ from the cascade reactions known from higher vertebrates (41). With the advent of cartilaginous and bony fishes, the adaptive immune system found its basic form including the ability to produce various classes of functional immunoglobulins. Although immunoglobulin-like sequences have been found in invertebrates, the high specificity and re-arrangement of V, D, and J domains associated with antibodies was first seen in these fish groups. The recombination activating genes RAG1 and RAG2 (RAGs) play a central role in this process and it is noteworthy that RAG-like sequence genes have been recognized in the early chordate *Amphioxus* (10). This adds to the notion that some immune-related genes in invertebrates and early chordates have had other functions before the adaptive immune system evolved.

## Signal Molecules

Coordination of cellular processes must be an integrated function even in the most primitive multicellular animals in order to maintain shape, structure, and function. Orchestration of complex reactions is carried out by various cytokines. Such molecules have been described in primitive invertebrates and although many of these may not be homologous to vertebrate cytokines, several studies have shown effects on the immune reactivity in invertebrates following stimulation with recombinant vertebrate cytokines. Thus, TNF-alpha, IFN-γ, and IL-8, have been demonstrated to induce reactions in worms, mollusks, and insects suggesting that these animals apply interleukin-like signal molecules (42–44). Earthworm coelomocytes responded to recombinant human IL-12 and IFN-γ by increasing phagocytosis (43) and Blue mussel hemocytes responded to TNF-α stimulation by increased stress reaction and decreased phagocytosis (42). Likewise, insect (fruitfly) cells were stimulated by recombinant human IL-8, which is associated with increase of phagocytic cells and subsequent expression of insect cytokines upd-3 and dhf (44). However, based on the fact that corresponding genes have not yet been described in these invertebrates it must be framed that these results should be observed with some caution.

However, some cytokines have been found encoded in the genome of certain invertebrates. A central regulating molecule is TGF-β, which may secure moderation of inflammation and initiate and sustain repair functions. It belongs to a family with numerous members in mollusks, nematodes, insects, echinoderms, and tunicates. Even the genome of cnidarians represented by the sea anemone *Aiptasia pallida* contains genes encoding TGF-β, and it was demonstrated experimentally that this cytokine depressed immune reactions including nitric oxide production (45). Another central cytokine is the macrophage migration inhibitory factor (MIF), which was released following infection with the digenean trematode parasite *Schistosoma mansoni* (46). MIF has also been described from the Pacific white shrimp *Litopenaeus vannamei* in which it functions as a prominent pro-inflammatory cytokine, which is up-regulated following viral infections (47) and predominantly expressed in blood cells, heart, and hepatopancreas. The Pacific oyster genome encodes an IL-17 like cytokine, which is highly expressed following injection with pathogenic bacteria (48). The cytokine allograft inflammatory factor-1 (AIF-1) has been described from the same host (49). It was found to stimulate phagocytic activity of oyster granulocytes. Crustaceans such as the freshwater crayfish produce a series of astakine cytokines (50–52), which have impact on hematopoiesis. The Chinese mitten crab produces suppressors of cytokine signaling (SOCS2) in various cells and organs following challenge with pathogenic bacteria (53). Fruitflies produce various cytokines including helical cytokines (44). In more developed invertebrates (echinoderms) (33) and primitive chordates (8), corresponding signal molecules have been described. The LPS-induced TNF-α factor (LITAF) gene was recently detected in *Amphioxus* (8) where it functions not only as a transcription factor for expression of TNF-α but also may be regulating innate responses in general. In lampreys, one of the most primitive vertebrates, a tumor protein homolog has been found to regulate cytokine secretion from various leukocytes (54). Our knowledge within cytokine evolution has recently been expanded particularly with regard to fish. Thus, IL-1, IL-2, IL4/13, IL-6, IL-7, IL-8, IL-10, IL-12, IL-13, IL-15, IL-17, IL-21, IL-22, IL-25, and IL-35 have been recognized in bony fish (55) and corresponding arrays might be expected to occur in cartilaginous fish (sharks and rays) (22). It should be framed that although sequence similarities suggest that lines of development from primitive animals (invertebrates and chordates) to higher vertebrates exist, one should be open for change of function of gene products during evolution. Thus, regulation of cellular communication may apply different cytokines at different stages even for corresponding functions.

## Evolution of Macrophage Function

Macrophage function in a higher vertebrate host organism may be directed along different pathways characterized as M1 and M2 functions (56). These lines are specialized in “Fight” or “Repair” systems, respectively, related at least partly to cellular use of Arginine. This amino acid can be converted to nitric oxide (NO) by inducible nitric oxide synthase (iNOS) or to Ornithine and Urea by Arginase. The former reaction (previously termed classical activation) makes the macrophage capable of fighting and killing invading microbes by use of the reactive N-species whereas the latter (alternative activation) can be characterized as the repairing pathway. In higher vertebrates, M1 and M2 cells are associated with expression of different cytokine profiles but it cannot be excluded that these two phagocyte functions are regulated in a special way in invertebrates. Evidence has been produced that this division of macrophage function may occur in fish. In rainbow trout putative macrophages (MHCII positive cells) are found widespread in various tissues even in the early yolk sac larva (57). Infections with *Ichthyophthirus multifiliis* (a ciliated skin and gill parasite) elicit expression of iNOS in rainbow trout (38, 39) and *Myxobolus cerebralis* infection lead to iNOS or Arginase-2 expression dependent on the susceptibility of the rainbow trout strain used (58). In addition, salmon louse *Lepeophtheirus salmonis* infections of Atlantic salmon skin was associated with an upregulation of the arginase gene (59). A related switch from a Th1 to a Th2-like reaction in rainbow trout skin infected with flagellates was recently described (60). So although M1 and M2 differentiation has not yet been detected in invertebrates, at least fish seems to have developed arginase, which makes M1 and M2 differentiation possible. Thus, Arginase is found in only one form in micro-organisms and invertebrates, a form which is not related to the ornithine–urea cycle, whereas fish may possess the necessary enzymes (61).

## Connecting Innate and Adaptive Responses

It was with the appearance of the vertebrates that a higher degree of immunological sophistication (adaptive immunity) was evolved. Vertebrates developed the MHC, T-cell receptors, and immunoglobulins as an additional weapon and regulatory system. The most primitive fish (agnathans such as hagfish and lampreys) possess special lymphocyte receptors composed of leucine reach repeats suggesting that this group followed a divergent line of development. With the cartilaginous fish (sharks and rays) and bony fish, immunoglobulins appeared. Some modern fish today carry at least three classes of immunoglobulins [IgM, IgT (Z), IgD], an array, which has been further developed in amphibians (IgM, IgX, IgY, IgD, IgF), reptiles (IgM, IgY, IgA, IgD), birds (IgM, IgY, IgA, IgD), and mammals (IgM, IgG, IgA, IgD, IgE) (62). Despite the lack of these specialized proteins (immunoglobulins) in lower vertebrates and invertebrates, this does not mean that immunity is less well developed in primitive animals. In fact, a rich array of innate immune genes and high variability of innate effector molecules provide animals such as earthworms, snails, mussels, shrimps, and insects with a capability to combat continuous attacks from microbes in their environment. Although central parts of these innate immune mechanisms present in invertebrates are conserved in higher vertebrates, it seems that the variability and diversity is much higher among invertebrates whereas higher vertebrates by fine-tuning the adaptive components (Igs, TCR, MHC) reach the same goal of clearing pathogens from the host organism. It may be hypothesized that the efficacy of the adaptive weaponry has allowed vertebrates to reduce the often impressing variety of innate effector molecules, which was available in earlier lineages.

## Coevolution of Parasite and Host as and Additional Driver of Innate Immunity Variation

It is evident that ancestors of existing pathogens have been able to evade innate and adaptive host immune mechanisms. Thus, immune reactions against viral, bacterial, and parasitic pathogens are in most cases only partly effective with regard to elimination of the intruding or established parasite in the vertebrate host (63, 64). It is a characteristic trait of both protozoan and metazoan parasites that the pathogens are able to deal with extensive cellular and humoral elements of the host immune system, a trait, which is securing parasite survival for extended periods (65). Coevolution of hosts and parasites has resulted in a tight interaction between innate and adaptive immune elements in the host and a rich but, to a certain extent, unexplored array of immune evasion mechanisms in the parasites. Also bacteria and virus apply an intricate system of immune evading mechanisms during invasion in order to survive host defenses (66, 67). Consequently, hosts may only survive, reproduce, and contribute to evolution by exhibiting new and more efficient immune molecules. This never ending arms race may be speculated to be at least partly responsible for the presence in modern times of an immense number of both hosts and parasites (68). However, in order to understand the principles of parasite immune evasion in higher vertebrates, including humans, it may be speculated that the basis for evasion will be found primarily in primitive invertebrates (16). Secondarily, we may trace it in the oldest and most original hosts possessing an adaptive immune system (12).

## Conclusion

Immune factors and recognition systems involved in differentiation of self from non-self may have been an integrated part of animal physiology since multicellular animals developed more than 600 mya. These innate mechanisms differ from the MHC system arising with the vertebrate lineage. Receptors, ligands, and signal molecules may initiate relevant actions by use of a series of effector molecules, which lead to elimination of pathogens or re-establishment of the injured tissue in the individual. These basic elements have been found even in sponges and cnidarians, two ancient invertebrate groups. The immune molecules and cellular products involved in these reactions are encoded by genes, which have similarities with elements even in higher vertebrates. Mollusks, crustaceans, insects, and echinoderms make use of cytokine like molecules resembling TGF, MIF, TNF, and interleukins. In addition, receptor molecules (TLRs), complement, and immunoglobulin-like sequences are being used by these invertebrates for various purposes. However, it is likely that although many immune genes and effector molecules can be found in the early invertebrates, their mode of action may differ considerably from the corresponding reactions in vertebrates. It is even likely that genes encoding factors with non-immunological roles in invertebrates may be used for immunological purposes in higher vertebrates, and vice versa. The dramatic environmental events on the geological time scale, with several periods of climate changes and extinction of major animal groups, have created a basis for selection of a multitude of new variants. Interactions with pathogens, which continuously are developing immune evasion mechanisms in their encounter with the host immune system, may further stimulate the never ending evolution of the immune system. The phagocyte function, taken by macrophages in vertebrates, has also been present in the earliest invertebrates. Corresponding cells have reached increasingly sophisticated levels during invertebrate evolution, and in vertebrates they exhibit high diversity. These cells have, in vertebrates, been equipped with MHC II molecules, which make them indispensable partners for B- and T-lymphocytes. They have obtained the ability to produce and communicate through an extensive cytokine network and they seem to be able to take a fight or repair function on their own reactions, which were seen also in the early invertebrates. In brief, immune reaction building blocks are ancient and appeared at various stages during evolution. Some were lost, some were moderated, and some even obtained another function during evolution. When adaptive immunity evolved with the vertebrate lineage, the old and still existing elements were further incorporated in the new hosts for optimization of immunity under the new conditions.

## Conflict of Interest Statement

The author declares that the research was conducted in the absence of any commercial or financial relationships that could be construed as a potential conflict of interest.
